# Underlying mechanism of hemodynamics and intracranial aneurysm

**DOI:** 10.1186/s41016-021-00260-2

**Published:** 2021-12-01

**Authors:** Haishuang Tang, Qingsong Wang, Fengfeng Xu, Xiaoxi Zhang, Zhangwei Zeng, Yazhou Yan, Zhiwen Lu, Gaici Xue, Qiao Zuo, Yin Luo, Jianmin Liu, Qinghai Huang

**Affiliations:** 1 Department of Neurosurgery, Changhai Hospital, Naval Military Medical University, 168 Changhai Road, Shanghai, 200433 People’s Republic of China; 2 Naval Medical Center of PLA, Naval Military Medical University, Shanghai, 200050 People’s Republic of China; 3 Department of Cardiology, the First Medical Centre, Chinese PLA General Hospital, Beijing, 100853 People’s Republic of China

**Keywords:** Hemodynamics, Inflammation, Atherosclerosis, Intracranial aneurysm

## Abstract

In modern society, subarachnoid hemorrhage, mostly caused by intracranial aneurysm rupture, is accompanied by high disability and mortality rate, which has become a major threat to human health. Till now, the etiology of intracranial aneurysm has not been entirely clarified. In recent years, more and more studies focus on the relationship between hemodynamics and intracranial aneurysm. Under the physiological condition, the mechanical force produced by the stable blood flow in the blood vessels keeps balance with the structure of the blood vessels. When the blood vessels are stimulated by the continuous abnormal blood flow, the functional structure of the blood vessels changes, which becomes the pathophysiological basis of the inflammation and atherosclerosis of the blood vessels and further promotes the occurrence and development of the intracranial aneurysm. This review will focus on the relationship between hemodynamics and intracranial aneurysms, will discuss the mechanism of occurrence and development of intracranial aneurysms, and will provide a new perspective for the research and treatment of intracranial aneurysms.

With the development of modern society, cerebrovascular disease has gradually become a major threat to human health, causing serious economic burden to families and society. Intracranial aneurysm (IA) is one of the most common cerebrovascular diseases, and one epidemic study revealed that the prevalence of IA among 35 to 75 years reached 7.0% (5.5% for men, 8.4% for women) in China [1]. One IA ruptured, the subarachnoid hemorrhage (SAH) results in serious accidents, and less than one third patients will lead to normal life [2]. Currently, the clinical strategies in treating IA are mainly restricted to surgical clipping and endovascular therapy. Although huge progress in IA treatment has been made, the complications and economy burden of both methods, especially when IA ruptured, cannot be ignored.

Till now, the etiology of IA remains to be clarified; the genetic aspect, living habit, environment, and estrogen hormone are all reported to be closely related to IA development [3–5]. Among the potential risk factors of IA, abnormal hemodynamics is one of the location-specific dangerous factors, which show high relevance with vessel inflammation and atherosclerotic plaque [6]. At present, many studies have confirmed that abnormal hemodynamics, as an initiating factor, is involved in the occurrence and development of IA [7, 8]. This review will focus on the pivotal role between hemodynamics and IA, targeting to provide novel perspective in IA research and clinical treatment.

## Overview of hemodynamics in cerebral vessel

In human vascular system, the state of blood flow can be divided into laminar flow and turbulent flow according to its velocity and geometrical parameter. Laminar flow is mainly distributed in the vertical vessels with large diameter, while turbulent flow is mainly distributed in the small and curved vessels [9]. Under the turbulent state, the blood flow velocity is relatively high and the geometrical parameter is more complex, which causes mechanical force to the blood vessel wall. Under the continuous abnormal blood flow stimulation, the function of the endothelial cells is disordered and becomes the initiating factor of IA development [10].

The mechanical force of blood flow on the tube wall mainly include the stretch of endothelial cells, the impulse force on the tube wall, and the tangential force between the blood flow and the tube wall. The tangential force between blood flow and wall follows the Poiseuille’s law, which is also called wall shear stress (WSS), calculated by in Pascal (PA) or Newton/square meter (N/m^2^). It reflects the friction between blood laminar flows with different flow velocities in the blood vessel. Therefore, WSS can also be regarded as the friction formed when blood flow contacting with the blood vessel wall. In physiological state, the average value of WSS is about 15dynes/cm^2^ in human large blood vessels, while in fact the value of WSS of different parts of blood vessels is significantly various because of the complex distribution of blood vessels in human body. In the common carotid artery, WSS ranges from 9.5 to 15 dynes/cm^2^, while in the femoral artery, WSS ranges from 3.9 to 4.9 dynes/cm^2^ [11, 12]. In the vessels with large diameter and regular morphology, WSS keeps in a stable physiological state, while in the vessels with curved or bifurcated vessels, the abnormal WSS leads to continuous stimulation to the vessels, destroying the integrity of the vascular structure, which triggers the activation of inflammatory cells and local inflammatory reaction [13].

Different from other organs, the brain accounts for 20% of circulating blood volume. Meanwhile, the anatomical of cerebral artery is much more complicated, especially the curves and bifurcations in Willis circle [14]. To better investigate and simulate the hemodynamic situation in cerebral artery, computational flow dynamics (CFD) was wildly applied in vessel visualization and measurement [15]. Other for WSS, CFD can also provide with oscillating shear index (OSI) which reflect the vessel wall resilience. Geometric simplification of cerebral vessel by CFD makes it possible in assessing the complex blood dynamics via noninvasive approach, computing the blood quantity and distribution in different areas [16].

## Hemodynamics and intracranial aneurysm

**Fig. 1:**
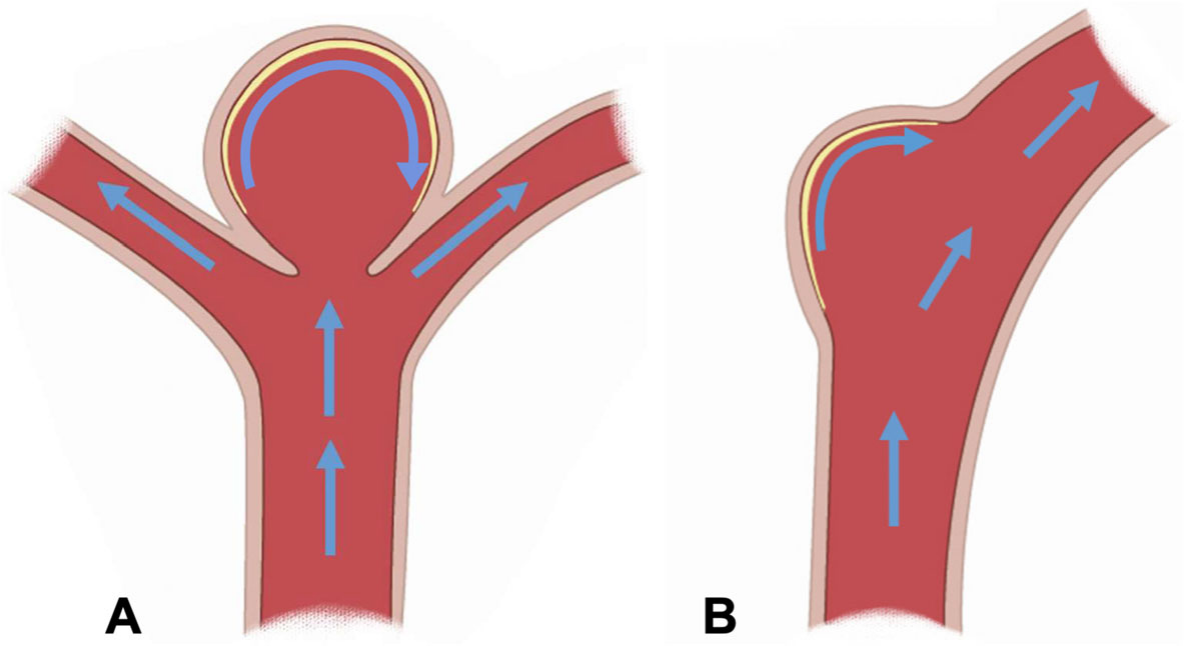
Two identical location of intracranial aneurysm in the brain. The two identical location of intracranial aneurysm in the brain showing the relationship between hemodynamic and intracranial aneurysm; the atherosclerotic plaque is usually companied with intracranial aneurysm. **A** Intracranial aneurysm located in vessel bifurcation. **B** Intracranial aneurysm located in vessel sidewall

Hypertension, smoking and drinking, environmental factors, and genetic factors are all reported to be risk factors of intracranial aneurysm. At present, many studies evidenced that abnormal hemodynamics is closely related to the occurrence and development of intracranial aneurysms [10, 17]. Animal aneurysm models are necessary for clinical device testing and basic researches, and animal models using hemodynamic induced aneurysm method are wildly utilized for various purposes. Morimoto et al. introduced a mouse aneurysm models by ligating bilateral posterior renal arteries and left common carotid artery [18]. Tutino et al. created basilar terminus aneurysm in rabbits induced by ligating bilateral common carotid artery ligation in order to increase artery blood flow [19]. Other studies also used vessel anastomosis technique to create aneurysm models, which further confirmed the close relationship between IA development and hemodynamics [20, 21] (Fig. 1).

The Willis circle was the most frequent place of IAs development owing to its special anatomy structure, which is reported to be closely related to irregular hemodynamic stimulation. Signorelli et al. reported that about 90% of saccular IAs were located in Willis circle, and other IAs are mostly located in the vessel bifurcations [8]. Willis circle is an important collateral circulation in the brain, which plays a pivotal role in connecting the blood circulation of both anterior with posterior hemispheres and the left with right hemispheres. Kapoor et al. suggested that in autopsy examination, the integrity of Willis can only be seen among 40% of the population, and anatomy variation of Willis circle exists wildly in more than half of the people, resulting in the diversity and complexity of local intracranial blood flow [22].

Except for the congenital variation of Willis circle described above, many people undertake tissue abnormality in blood vessel owing to abnormal gene expression. Among these people, the aneurysms are more prone to occur under hemodynamic stimulation. Schmid et al. reported that elastin layer of the vascular wall in intracranial aneurysms is often missing, which may be related to the abnormal gene expression of the structural protein of the vascular wall in human body [23]. Torres and Harris found that in patients with polycystic kidney disease, 2 to 10% of patients suffer from aneurysms, and these people are unable to encode a certain vascular structural protein [24]. In patients with Marfan syndrome, intracranial aneurysms were developed owing to lack of gene that can encode collagen [25]. Olubajo et al. revealed that among people who suffer from Ehlers-Danlos syndrome, the type III collagen gene mutation increases the arterial fragility, and IAs were one of the most clinical manifestations among these patients [26].

To further elucidate the causes of IA location in brain artery, many researches focused on the correlation between IA location and WSS level. Meng et al. created new branch points in the carotid artery in dogs, under high WSS and high WSS gradient environment, the arterial wall presented with destructive remodeling around the irregular flow, resembling the process of IA initiation [27]. Gao et al. created rabbit aneurysm models by carotid artery ligation, increasing the regional blood flow. They evidenced that all rabbits in model group formed nascent aneurysms under high WSS condition and further histology examination evidenced the aneurysms share similar characteristics with absent internal elastic lamina and thinned media layer [28].

In the further development of aneurysms, researchers believe that longitudinal blood flow impinging on the vessel wall is an important risk factor in aneurysm growth, and continuous blood flow further destroys the structure of the vascular wall [29]. However, there are also studies showing that low WSS stimulation is closely related to the progression of aneurysm [30]. Another study conducted by Machi et al. revealed that low WSS and high OSI promoted IA growth, and in their study, they clarified that the areas of IA growth showed low shear conditions with increased oscillations at the site of growth [31]. The low WSS condition formed by blood flow is similar to the environment forming atherosclerotic plaque, which further explains that atherosclerotic lesions are often found in the anatomy of IA. At the same time, low WSS promotes vascular inflammatory response and endothelial cell dysfunction and gradually destroys the entire structure of the vascular wall, which becomes an important basis for further development of IA. One meta-research summarized the CFD studies targeting at the relationship between IA and WSS; they reported that high WSS was relevant to IA initiation while low WSS contributed to IA rupture [32]. Till now, although the consensus was not reached about the exact relationship of IA growth or rupture with the WSS level, the joint perspective could be summarized that low WSS and high WSS jointly exerting effect in the natural history of IA [33].

## Hemodynamic stimulation and vessel inflammation

Vessel inflammation was the starting event in IA development. In normal condition, the cerebral artery consists of 3 layers: the intima layer, the medial layer, and the adventitia layer. The integrity of vessel structure enhances the protective effect against risk factors. The WSS produced by the stimulation of blood flow is initially felt by the endothelial cells, and vitro study has revealed the abnormal function of endothelial cells under irregular WSS [34]. Gene transcription in endothelial cells under the WSS environment are in a time- and space-related manner; the same conditions are also applied in the expression of ion channels on the surface of the cell membrane and the expression of cytoskeleton protein. Under physiological conditions, nitric oxide (NO) was generated mainly by endothelial nitric oxide synthase (NOS) in endothelial cells and exerts cardioprotective role by inhibiting platelet aggregation, inhibiting the proliferation of smooth muscle cells which can relax blood vessels, and inhibiting the expression of inflammatory factors [35, 36]. In endothelial cells, NO is continuously synthesized by endothelial nitric oxide synthetase (eNOS). Paschoal et al. reported the activation of eNOS in endothelial cells is positively related to the size of WSS [37]. When the blood flow velocity suddenly increases and the blood flow morphology is unstable, the increase of WSS is accompanied by the increase of eNOS activity and NO expression, and this effect is mediated by MAPK signal pathway phosphorylation [38]. As mentioned above, the increase of NO secretion in high WSS environment exerts a protective effect on blood vessels, which contradicts the theory that high WSS stimulates the formation of intracranial aneurysms. In fact, in the initiation of aneurysm, the blood flow change is a continuous and slow process rather than a transient change of blood flow. Under the long-term mechanical stimulation of blood flow, endothelial cells change their morphology and function and then gradually lead to apoptosis, and then, the activity and quantity of eNOS in the cells decrease, so as quantity of NO secretion [39].

In addition to eNOS, there are also inducible nitric oxide synthases (iNOS) in endothelial cells. Its main function is to produce iNOS when endothelial cells suffer from injury and excessive inflammation stimulation. Study suggested that iNOS was not directly related to the occurrence of IA, but may play an important role in IA progression and size growth. In iNOS knockout mice, compared with wild-type mice, the incidence of aneurysms in iNOS knockout mice was not significantly different, but the diameter of aneurysms was significantly reduced [40].

However, when the endothelial cells are unable to adapt to the hemodynamic stimulation, the inflammatory molecular expression is repressed. Wang et al. found that in dogs, high WSS was associated with endothelial degeneration, which further reduce the expression of inflammatory molecule induced by endothelial cells. In their study, the interleukin-1beta (IL-1β), matrix metalloproteinase-2 and matrix metalloproteinase-9 (MMP-2, MMP-9), and nitric oxide synthetase (NOS) in aneurysm wall were all decreased compared to normal artery [41].

**Fig. 2:**
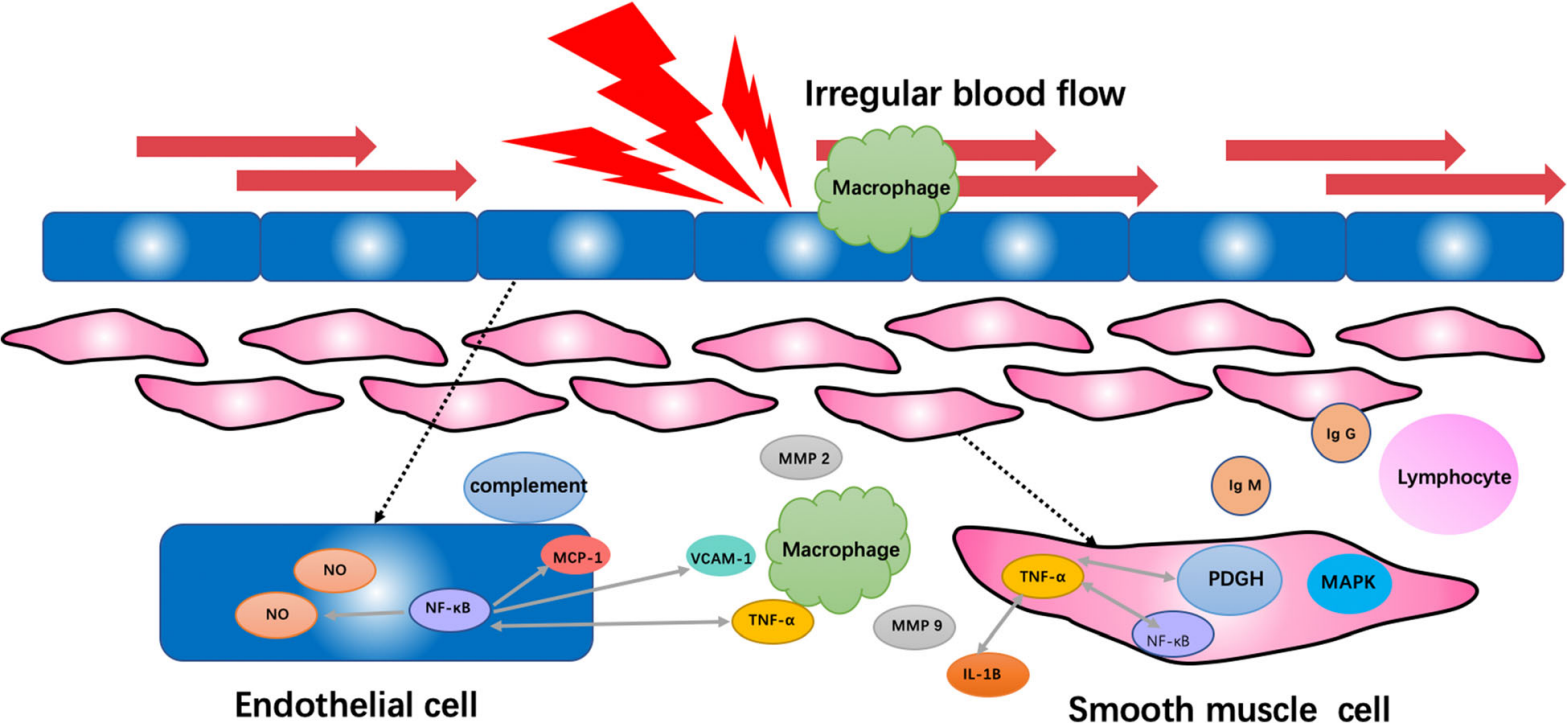
Hemodynamic induced inflammatory process in intracranial aneurysm development. The irregular blood flow triggers the inflammation in the artery, and the inflammatory cytokines including IL-1β, MMP-2, MMP-9, MAPK, PDGH, NF-κB, and NO collaboratively contribute to the development of intracranial aneurysm

Except for the hemodynamic induced NO secretion, other inflammatory molecules are also reported to be associated with blood flow shear stress. Sumpio et al. pointed out that mitogen-activated protein kinase (MAPK)-mediated signaling pathway in endothelial cells plays an important role in detecting shear stress and triggering intercellular signals [42]. Eng et al. reported that the activation of nuclear factor-kappaB (NF-κB) and platelet-derived growth factor (PDGF) changed according to WSS level, and under low level WSS environment, the expression of NF-κB and PDGF were repressed [43] (Fig. 2).

## Hemodynamics and cerebral atherosclerosis

Atherosclerosis can be seen as the chronic inflammation of artery owing to lipid dysmetabolism [44]. The lipid overload, especially low-density lipoprotein and oxidized low-density lipoprotein, triggers the dysfunction of endothelial cell. The following activated monocyte and macrophage further promote the transformation of foam cells from smooth muscle cells [45]. The pathophysiology of atherosclerosis accounts for the primary etiology of various vascular diseases, such as ischemic stroke, myocardial infarction, and coronary aneurysm [46–48]. Many studies revealed the close relationship between atherosclerosis and IA. Hashimoto et al. reported that unruptured aneurysm wall enhancement was corresponded atherosclerotic lesions detected by magnetic resonance angiography [49]. The similar finding was also evidenced in other studies [50, 51].

In the cerebrovascular system, the bifurcation of common carotid artery is the most common site of atherosclerosis. Near the proximal end of the internal carotid artery, there is a swollen part of the blood vessel, which is called the carotid sinus. In the position of carotid sinus, the cross-sectional area of the blood vessel is about twice that of the distal internal carotid artery, which determines the instability of blood flow in the blood vessel [52]. In physiological state, the blood flow velocity is the largest in the center vessel while the velocity close to the wall of the blood vessel is lower, and the flow velocity is inversely proportional to the diameter of the blood vessel. When the common carotid artery is divided into internal carotid artery and external carotid artery, the flow velocity at the bifurcation site is the largest. Meanwhile, due to the presence of carotid sinus, the flow velocity of blood flow at the internal wall and external carotid artery is relatively high, while the flow velocity of blood flow at the external wall of carotid sinus is the lowest [53]. What is more, due to the existence of curved structure of blood flow, the local circulation of blood flow is formed in the carotid sinus. These factors together determine that atherosclerosis are more prone to initiate in the lateral wall of the carotid sinus [54].

From the perspective of hemodynamic, the anatomy results reveal that atherosclerosis prone to lateral side of the arterial wall and the bifurcation of the artery was also related to low level of WSS [53]. Zhang et al. found that in the process of atherosclerosis development, the decrease of WSS level was negatively associated with vessel wall elasticity; thus, the analysis of WSS can reflect the atherosclerosis development, which can further reveal the location of IA initiation [13]. Under the continuous of irregular WSS stimulation, endothelial cells change into a circular state to reduce mechanical stimulation. When the stimulation surpasses the protective effect, the dysfunction of endothelial cells damages the integrity of vascular endothelial barrier, providing opportunity for low-density lipoprotein particles entering the vascular wall, which is also a typical pathological feature of atherosclerosis [55].

The process of atherosclerosis development is dynamic, so as the vascular structure. This change is simultaneously companied with local hemodynamic alteration of blood vessels. One of main results of atherosclerotic lesion is vascular stenosis. When the vascular stenosis reaches a certain degree, the blood flow velocity increases, so as the regional WSS. High level WSS not only slows down the pathological process of atherosclerosis, but also increases the risk of atherosclerotic plaque falling off, which has become a major risk factor for acute ischemic stroke [56]. Wityk et al. reported that the atherosclerosis-induced cardiovascular diseases are particularly prominent in Asian population, although there is still lack of exact explanation for this phenomenon [57]. In addition, artery stenosis caused by atherosclerosis not only brings high blood flow status in the stenosis site, but also brings disorder in the downstream of the stenosis site. The blood flow of different flow velocity and direction is mixed together, which further promotes the formation of atherosclerotic plaque.

It should be noted that the anatomical variation of carotid artery is common in the population; meanwhile, the anatomical characteristics of cerebral artery are also affected by age, gender, and other factors. In addition to the effect of stenosis on hemodynamics, the angle of bifurcated vessels is also a pivotal factor affecting hemodynamics. At the junction of the vertebrobasilar arteries, the two vertebral arteries converge to form the basilar artery. From the perspective of hemodynamics, this vascular anatomical feature has many similarities with the bifurcation of the common carotid artery. It has been confirmed that WSS is lower at the junction of vertebrobasilar artery and the lateral part of basilar artery, which are also prone to developing atherosclerosis lesions [58].

In the location where internal carotid artery enters the skull, the siphon segment blood vessels become S-shaped, which determines the complexity of hemodynamic characteristics of blood flow [59]. In the curved vessels, the low WSS is usually located in the medial wall of the artery, while the high WSS is usually located in the lateral wall of the artery, while the siphon segment contains two curved vessels, so it is difficult to judge the WSS level in this artery. In addition, the Willis circle varied widely in the population, thus the complex hemodynamics lying in this area. Under such condition, CFD is used to simulate the structure of cerebral blood vessels and the characteristics of blood flow aberrations. CFD results combined with the anatomy of intracranial blood vessels provide favorable basis for the prediction and evaluation of cerebral atherosclerosis [60].

## Summary

The structure of cerebrovascular is complex, determining the variation of blood flow. Under physiological condition, the stable blood flow supplies energy and oxygen to brain tissue via cerebral vessel. The imbalance of blood flow and vessel wall results in IA initiation and even SAH caused by IA rupture. Many efforts have made to elucidate the IA etiology, and much progress has been made in clinical treatment. Although many researches have shed light on the strong relationship between hemodynamics and IA, the exact mechanism during the IA initiation and progression is still lacking. In our review, we discuss the correlation between the two aspects. The continuous irregular blood flow stimulation damages the integrity of the vessel wall, triggering the inflammatory progress. The chronic inflammation further provides an ideal environment for the formation of atherosclerotic plaque. The atherosclerosis and inflammation jointly promote IA occurrence and development.

Our review summarizes the previous published researches of hemodynamic role in IA; several limitations still exist in this review. IA initiation and growth are dynamic, and all the studies described focused on one certain stage of IA, lacking long-term dynamic outcome. Another limitation is that the blood flow simulated via CFD is oversimplified to some extent, which is unable to mimic the complex flow condition perfectly in the brain artery. At present, the research on hemodynamics and cerebrovascular diseases is still limited. The technique progress of CFD and imaging technology will provide a novel perspective for hemodynamic research on IA, which brings hope to promote IA clinical treatment.

## Data Availability

The datasets used and analyzed during the current study are available from the corresponding author on reasonable request.

## Abbreviations

IA: Intracranial aneurysm
SAH: Subarachnoid hemorrhage
WSS: Wall shear stress
CFD: Computational flow dynamic
OSI: Oscillating shear index
NO: Nitric oxide
NOS: Nitric oxide synthase
eNOS: Endothelial nitric oxide synthetase
iNOS: Nitric oxide synthases
IL-1β: Interleukin-1beta
MMP-2: Matrix metalloproteinase-2
MMP-9: Matrix metalloproteinase-9
NF-κB: Nuclear factor-kappaB
PDGF: Platelet-derived growth factor
